# Oil-Based Delivery Control Release System Targeted to the Later Part of the Gastrointestinal Tract—A Mechanistic Study

**DOI:** 10.3390/pharmaceutics14050896

**Published:** 2022-04-20

**Authors:** Lingping Zhang, Marie Wahlgren, Björn Bergenståhl

**Affiliations:** Department of Food Technology, Engineering and Nutrition, Lund University, 221 00 Lund, Sweden; marie.wahlgren@food.lth.se (M.W.); bjorn.bergenstahl@food.lth.se (B.B.)

**Keywords:** oil-based delivery system, in vitro method, later part of the small gastrointestinal tract

## Abstract

Oil-based drug delivery systems have been studied in different aspects. The present study proposes a new application for an oil-based delivery system, focusing on controlled release until the drug reaches the later part of the small intestine. Bulk surfactants and interfacial surfactants were added into the oil formulation to provide a better mechanistic understating of the lipolysis. Validation of the modified in vitro method shows the overall conversion from medium-chain triglyceride oil (MCT oil) to free fatty acids (FFA) of 100 ± 4% in five replicates. This fully converted level and high reproducibility are fundamental for the following investigations where any retarding effect can be distinguished from the experimental errors. The results show that viscosity and thermodynamic activity have limited retardation. Furthermore, the former may change the kinetics of lipolysis, while the latter changes the equilibrium level. The gel-forming retarder (ethylcellulose) displayed a strong effect. Whereas the lipolysis was significantly retarded (>50%) when the retarders altered the interfacial composition (poloxamer 407), degradable interfacial surfactants did not have the same effect. However, surface-active, lipolysis-resistant retarders with a high CMC did not show a retarding effect.

## 1. Introduction

Oil-based oral drug delivery systems have been studied for a wide range of applications [1,2,3,4]. They have primarily been explored for poorly water-soluble drugs with the aim of increasing the solubility and bioavailability of these drugs [5,6]. However, oil-based systems have additional advantages which make them interesting for a wide variety of active pharmaceutical ingredients (APIs), allowing, for example, the formulation for low water activity systems; therefore decreasing the usage of excipients (e.g., stabilizers and preservatives). Moreover, the system stability may also be increased during manufacturing and storage. Overall, the oil-based systems are expected to be relatively simple and robust.

In this study, we are trying to understand the degradation of oil-based systems, for which the target location is the later part of the small intestine and colon. Such local targeting system has been applied to treat local diseases of the large intestine such as inflammatory bowel disease (IBD) and colon cancer and is suitable for chronotherapy and systemic drug delivery for other diseases, as well as a possible system for probiotics [7,8,9,10]. The physiological challenges of targeting the later part of the small intestine or colon include primarily large variations in the time of passage for a drug product through the gastrointestinal tract (GI) tract, large pH variety (1.5–7), and the effects of different secretions from the digestive system before reaching the colon (enzymes, bile acids, ions) [11]. Different formulations have been developed to tackle these challenges, such as enteric coatings, bacteria-triggered systems, pH-triggered systems, time-based delivery, or different combinations [12,13,14,15]. However, these releasing triggers are designed using the physical conditions of a healthy person, and have a high risk of not working in diseased conditions [16]. Thus, our aim here was to develop an oil-based control release system that possibly could be less affected by the physiological conditions of the GI tract.

The concept applied is that a specific component, a retarder, can be added to delay oil digestion and allow the system to survive until the drug reaches the later part of the small intestine or the colon. Oil digestion, also termed lipolysis, is defined as the degradation of triglyceride to free fatty acids (FFAs) by the enzymatic hydrolyses. During lipolysis, different enzymes (e.g., lipase and colipase) and bile salts play the most important role [17,18]. Adding different components to oil formulations to control the digestion of triglycerides has been extensively studied, especially to tackle the growing global diabetic issues [19,20,21,22]. The components that successfully inhibit most lipolysis covalently bind to the lipase, which causes other side effects [23]. Therefore, substantial efforts have been put into finding alternative methods. So far, preventing lipase from reaching the oil–water interface has proven efficient in retarding the lipolysis, where mainly water-soluble surfactants have been used (e.g., polysorbate 20 (Tween 20), polyethyleneglycol lauryl ether (Brij 35), sodium lauryl sulphonate (SDS), poloxamer 407) [24,25]. In previous research, these water-soluble surfactants were always compared within certain groups, e.g., non-ionic surfactants, small molecular surfactants, polymeric surfactants, etc. Additionally, the viscosity of the oil phase was also increased by using lipids with different chain lengths of the [26,27]. Overall, in this work, we aimed to comprehensively map the retardation mechanism in the oil-based control release system. A better understanding of the retardation mechanism is not only the prerequisite for developing a successful oil-based control release system, but it could also be a guideline for other oral delivery systems.

It is also essential to have a well-established in vitro model to evaluate such an oil-based control release system [28,29]. Different in vitro models with different complexities cover different aspects of digestion. Some in vitro models focus more on mimicking the upper GI tract’s chemical and mechanical properties (e.g., artificial stomach–duodenal model, TIM-1, and dynamic gastric model) [30,31,32]. This study explored the impacts of different secreted compounds during digestion. Pancreatic extract (including pancreatic lipase, colipase, phospholipase A2, etc.) and bile extract (mixture of different bile acids) with phosphatidylcholine (PC) were used instead of pure lipase or a certain bile acid, the concentrations of which preliminarily mimicked the fasted state in the small intestine. A modified in vitro model was achieved with high conversion from medium-chain triglyceride oil (MCT) to free fatty acids (FFAs) and high reproducibility using relatively simple mechanical settings so that the observed retarding effect can be distinguished from experimental errors.

By using this modified in vitro system, we compared different retardation mechanisms. Retardation was due to increased viscosity or a gel formation of the oil phase by adding different bulk retarders into the oil. The impact of the retarders on the thermodynamic activity was also evaluated. Retardation caused by altering the interfacial properties (composition and stability) was analyzed by adding different interfacial retarders with different molecular sizes and different ratios between hydrophobic and hydrophilic groups. In this part, the competition between interfacial retarders, bile salts, and pancreatic lipase was discussed. Furthermore, digestible retarders and non-digestible ones are compared.

## 2. Materials and Methods

### 2.1. Materials

Pancreatin (porcine, P-1625, lot: SLCF5908), bile extract (porcine, B-8631, Lot: SLBX1760), glyceryl tributyrate, poly(dimethylsiloxane) 25 cSt, 750 cSt(dimethicon), Trizma maleate (tris(hydroxymethyl)aminomethane maleate), polysorbate 80, ethylcellulose (viscosity 300 cP, 5% in toluene/ethanol 80:20, extent of ethoxylation: 48%), n-hexadecane, and poloxamer 407 were purchased from Sigma-Aldrich (St. Louis, MO, USA). Pentaethylene glycol monododecyl ether (C_12_E_5_, BL-5SY) was bought from Nikko Chemicals (Tokyo, Japan). Sodium chloride and calcium chloride dihydrate were bought from VWR chemicals (BDH Prolabo, Melbourne, Australia). Soybean phosphatidylcholine, glycerol monooleate, polyglycerol polyricinoleate (PGPR), and cetearylglucoside (a 1:1 mixture of cetylglucoside (C16) and stearylglucoside (C18)) were donated by Cargill (Hamburg, Germany), DuPont Nutrition and Bioscience (Aarhus, Denmark), AAK (Karlshamn, Sweden), and Croda (East Yorkshire, UK), respectively. The MCT oil used was a fractionated coconut oil (Miglyol 812, fatty acid composition: C_6_ 0.1%, C_8_ 60.9%, C_10_ 38.5%, C_12_ 0.4%, certificate of analysis, Condea, Hamburg, Germany) [33]. The water used was obtained from a Milli-Q-water purification system (Millipore, Burlington, MA, USA). Sodium hydroxide 1 mol/L in an aqueous solution was bought from VWR chemicals (Radnor, PA, USA). All other chemicals were of analytical grade.

### 2.2. Preparation and Characterization of Pancreatin Extract

The pancreatin suspension was prepared as described by [34], with some modifications. In short, 4.98 g of pancreatin powder was suspended in 35 mL of distilled water and mixed using a vortex mixer (Kebo-Lab, REAX 2000) at speed 9 for 30 s. The suspension was then centrifuged in an Allegra X-15R centrifuge (Beckman Coulter, Brea, CA, USA) for 7 min at 4000 rpm. Then, a 30 mL aliquot was withdrawn, and pH was adjusted to 7 before being used in the in vitro model. The pancreatic suspension was prepared within 15 min of the start of the experiment to avoid the loss of lipase activity.

The lipase activity of pancreatin was determined using a TBU assay as described by [35,36], with a minor modification. Tributyrin (2.5 mL) was dissolved in a 50 mL buffer containing 150 mM NaCl, 2 mM tris, and 1 mM Ca^2+^. The same titration pH (pH 7), temperature (37 °C), and pancreatin suspension concentration were used in in vitro simulations. Pancreatin activity was expressed as tributyrin units, where one TB unit equals 1 µmol of butyrin acid released per minute at pH 7 and 37 °C. The linear part of the conversion curve, where no substrate restriction is presented, was used to determine lipase activity. The lipase activity was obtained by:

(1) alipase [TBUg]=Vtitrand·ctitrandmsample

where *V_titrand_* and *c_titrand_* are the volume and molarity of titrant (1 mol/L NaOH), and *m_sample_* is the mass of the enzyme sample.

### 2.3. In Vitro Method

The retardation was investigated using a simulated small intestinal system representing the fasted state. This was in order to have a robust system, even though about one-third of the actual lipolysis reaction is in the stomach [37]. To optimize the stability of the in vitro method developed by Zangenberg et al. [34], we investigated the sensitivity of different concentrations of bile solution, buffer, and pancreatin solution, and the final recipe is shown in Table 1.

Bile extract and pancreatin extract from porcine were applied to increase the similarities of the actual biological complexity instead of using mixed bile salts and pure enzymes. The ratio between bile extract and phosphatidylcholine was 1:4 to keep it the same as in vivo [38]. The concentrations of different bile salts from bile extract were calculated using the data from Christensen et al. [33]. A high concentration of 15 mmol/L of calcium ions was applied to ensure the complexation of 100% of the released FFAs (Supplementary Data). Meanwhile, a high concentration titrant (1.0 mol/L NaOH) was used to avoid extra dilution.

The in vitro lipolysis was performed in a thermostatic pH-stat equipment. It was started by adding freshly prepared pancreatin suspension to a pre-emulsified mixture of MCT oil, bile solution, and buffer (high shear rotor-stator emulsification (Ultra Turrax, speed 4) (Ystral, Ballrechten-Dottingen, Germany)) in a thermostatic beaker (37 °C). The pH of both solutions was adjusted to 7 with 1.0 mol/L NaOH before the titration was initiated. A two-step titration procedure was followed; first, a 2 h pH-stat kept pH at 7, and then an endpoint titration up to pH 9. The static pH 7 is slightly higher than used in other investigations [39,40,41]. At this pH value, the C_8_ and C_10_ fractions of the triglycerides (more than 98% of the MCT oil) can reach the maximum lipolysis extent while keeping within the physiological pH range [40]. In line with other investigations, pH 9 is the endpoint pH value [41,42]. This back titration was executed to avoid underestimating the lipolysis extent in the pH-stat procedure [43].

In this study, 3 moles of FFAs were calculated instead because 2-monoglycerides spontaneously isomerize to 1/3-monoglyceride (1/3-MG), leading to subsequent lipolysis releasing a third FFA and glycerol, as shown by others [33,44]. The net consumption of titrant is calculated from the total titrant consumption and by subtracting the background:

(2) $$V_{net}\left[mL\right]=V_{total}-V_{background}$$

where *V_total_* is the total titrant (1 mol/L NaOH) consumed, including both two-step titrations. *V_background_* is the titrant consumption in a blank experiment having all components except the triglycerides.

The relative conversion of FFAs from triglycerides is calculated from the consumption of titrant:

(3) $$\varphi_{lipolysis}\left[no\;unit\right]=\frac{V_{net}\cdot c_{titrand}\cdot M_{TG}}{3\cdot m_{TG}}$$

where *c_titrant_* is the concentration of the titrant; *M_TG_* and *m_TG_* are the molecular mass (g/mol) and mass of the MCT oil, respectively; and 3 is the maximum number of FFAs that could be released. A modified Equation (3) was used for digestible retarders, i.e., PGPR, polysorbate 80, and monooleate.

(4) $$\varphi_{lipolysis}\left[no\;unit\right]=\frac{V_{net}\cdot c_{titrand}\cdot M_{substrate}}{3\cdot m_{substrate}}$$

where *M_substrate_* and *m_substrate_* are the average molecular weight (g/mol) and mass of the MCT oil and digestible retarders, respectively.

After these adaptions, an almost complete conversion of MCT oil to FFAs was achieved (100 ± 4%) among five replicates, indicating high reproducibility. Along with comparing the conversions from MCT oil to FFAs, the time point (t_50%_) was chosen as the parameter to compare different conversion speeds. T_50%_ is the time point when the corresponding conversion of an oil formulation is equal to 50% of the final conversion of pure MCT oil.

### 2.4. Oil Formulations Investigated

The same amount of hydrolysable oil (0.5 g) was used each time. All measurements were conducted on at least two freshly (same day) prepared samples. Data sets were analyzed using one-way ANOVA. Different oil formulations were pre-emulsified the same way as pure MCT oil, except for ethylcellulose gel. Ethylcellulose gel was cut into small cubes, with each side length being less than 2 mm.

#### 2.4.1. Bulk Retarders

Dimethicon 25 cSt, dimethicon 750 cSt, polyglycerol polyricinoleate (PRPG), and hexadecane (with concentrations of 16% and 32%) were added as the bulk retarders; PGPR also served as an interfacial retarder. Bulk retarders were directly added into the oil phase, followed by gentle mixing. Bulk retarders were first mixed with MCT oil in a relatively larger amount (in a total of 10 g) to minimize the mixing error.

#### 2.4.2. Ethylcellulose

Ethylcellulose gel was prepared using an adapted method proposed by O′Sullivan et al. [45]. Briefly, 10% 300 cP ethylcellulose was added into the MCT oil and stirred with a stainless-steel stirrer under gradual heating. After the sample reached the temperature of 160 °C, the heating was continued for additional 5 min to ensure the complete dissolution of the ethyl cellulose. The sample was then cooled down to room temperature and kept until the gel was formed. It was placed in the refrigerator overnight before the analysis.

#### 2.4.3. Interfacial Retarders

Monooleate, polysorbate 80, poloxamer 407, C_12_E_5_, and cetearyl glucoside (with concentrations counted on oil of 3 wt% and 6 wt%) were added as the interfacial retarders. Detailed information of these retarders is shown in Table 2. Converting these two concentrations to the concentration counted on aqueous phase resulted in 0.015 wt% and 0.03 wt%, respectively. Due to the low concentrations, the viscosity was similar in different samples (around 16 mPa.s^−1^). The interfacial retarders, except polysorbate 80, were added into the water phase at an elevated temperature using a water bath. Higher temperatures ensured the complete dissolution of monooleate (37 °C), poloxamer 407 (50 °C), and cetearyl glucoside (80 °C).

**Table 2:** Molecular mass and critical micelle concentration (CMC) of interfacial retarders monooleate, polysorbate 80, poloxamer 407, and cetearyl glucoside.

Interfacial Retarders	Molecular Mass (g/mol)	CMC * (wt ppm)
Monooleate	356	1.4
Polysorbate 80	1310	1.6
Poloxamer 407	12,600	35
C_12_E_5_	406	28
Cetearyl glucoside	404	0.40

* The CMC values of monooleate, polysorbate 80, poloxamer 407, and C_12_E_5_ are recalculated from [46,47,48,49]. The CMC value of cetearyl glucoside is given by the manufacturer.

### 2.5. Droplet Size

The size of the pre-emulsified formulation after high shear rotor-stator emulsification was measured by a laser diffraction system Mastersizer 2000 (Malvern Instruments, Malvern, UK) to control the total effective area of the interface. The sizing was verified by light microscopy using a camera (DFK 41AF02, The Imaging Source, Bremen, Germany) attached to the light microscope (Olympus BX50, Tokyo, Japan). Emulsions were added into the flow system (Hydro SMSM small volume wet dispersion unit) containing MilliQ water, and then pumped through the optical chamber where the measurements were conducted. The mixing speed was kept at 600 rpm, and the laser obscuration was maintained between 5% and 6%. The refraction index was set as the same as the pure oil phase (n = 1.45), as the concentrations of other chemicals are relatively low. The diameter is the average of three repeated measurements, resulting in an average diameter (d(4,3)) of around 10 μm (Supplementary Data), which is in the lower range of size distribution in vivo (1–50 μm) [50].

### 2.6. Interfacial Tension

The pendant drop technique was used to measure the interfacial tension of the oil–water interface (Teclis, Civrieux-d’Azergues, France). A syringe (total volume of 500 μL) with a Teflon-coated needle (Thermo Scientific, Waltham, MA, USA) was used. The Young–Laplace equation was applied to analyze the shape of the drop to determine the interfacial tension, drop volume, and interfacial area. Measurements gave references value of 71 ± 0.5 mN/m for the water–air interface and 26 ± 0.5 mN/m for the water–oil interface at 37 °C. All systems reached the semi-equilibrium plateau rapidly; after this, the interfacial tension was relatively stable with time (15 min).

### 2.7. Rheological Analysis

Rheological measurements were conducted in a rotational rheometer (Kinexus, Malvern, UK) at 37 °C. The apparent viscosity of all the formulations, except the ethyl-cellulose gel, was measured using a shear-rate controlled program at 37 °C with a shear rate range from 10 to 100 s^−1^. All measurements were carried out in duplicates.

### 2.8. Texture Analyzer

The hardness of the ethylcellulose gel was measured by a texture analyzer TA-XT2i (Stable Micro Systems, Godalming, Surrey, UK) using a 6 mm probe. Texture profile analysis (TPA) was performed with a penetration speed of 0.5 mm/s and a target distance of 20 mm. All parameters were calculated automatically using Exponent ver.6.1.15.0 (Stable Micro Systems, Godalming, Surrey, UK).

## 3. Results

In this study, the retarding effects were compared between different formulations using the modified in vitro method as described above. Three hypotheses were brought out, and the results were presented accordingly.

### 3.1. Hypothesis 1: Change in the Consistency of the Oil Phase Influences the Lipolysis

**Figure 1:**
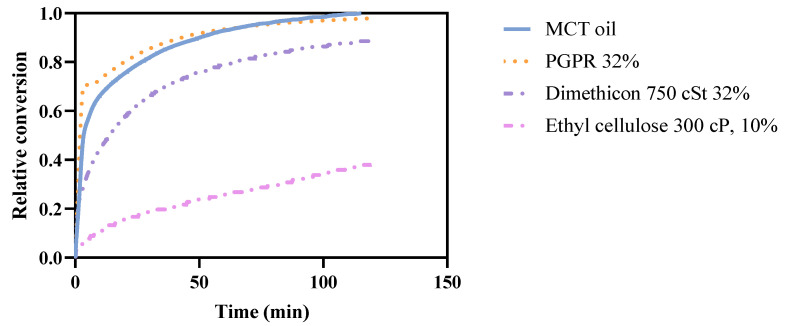
Relative conversion of MCT oil in the presence of different bulk retarders: PGPR 32%, dimethicon 750 cSt 32%, ethylcellulose 300 cP 32%. The curve is a mean value of duplicate measurements. The relative standard error of the method is 1.2%.

Two bulk retarders (dimethicon and PGPR) with two concentrations (16% and 32%, *w/w* counted on the oil) were added into the oil formulation to alter the fluidity of the oil phase to investigate if changing the consistency of the oil phase could influence the conversion of MCT oil during lipolysis. Ethylcellulose (300 cP, 10%), on the other hand, was added to form a gel network in the MCT oil. The viscosities of different formulations ranged from 13 mPa.s^−1^ (dimethicon 25 cSt, 16%) to 80 mPa.s^−1^ (PGPR 32%), corresponding to 0.81–5 times the viscosity of pure MCT oil, respectively (Table 3). The viscosity of ethyl cellulose was too high to measure. It forms a relatively firm and sticky gel and to characterize this gel we used a texture analyzer that gave a breakthrough hardness of 9.4 kPa. The in vitro results from the digestion of representative formulations are shown in Figure 1. Formulations with either dimethicon 25 cSt, dimethicon 750 cSt, or PGPR give a minor decrease in the conversion. With the exception of ethylcellulose (39% of total conversion), oil-soluble retarders have limited retardation on lipolysis (from 89% to 98% of total conversion). It is clear that only the highest viscosity formulation (32%, 750 cSt) has a significantly longer t_50%_ compared to others (Table 3), while t_50%_ of ethylcellulose is uncountable as it never reaches 50% conversion. On the other hand, PGPR has even shorter t_50%_ than pure MCT oil. Thus, a change in the viscosity may reduce the rate of degradation, but not at the final level of degradation, a gel-like semi-solid consistency significantly reduces the degradation rate.

### 3.2. Hypothesis 2: Thermodynamic Activity of Oil Phase Influences the Lipolysis

**Figure 2:**
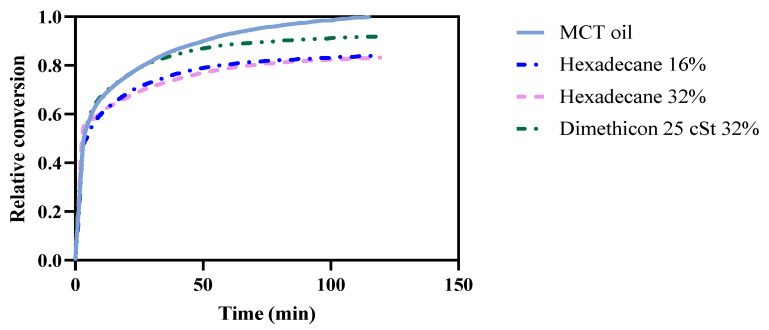
The relative conversion curve of hexadecane 16% and 32% and pure oil.

To evaluate the role of the thermodynamic activity of a nondegradable, low-viscosity retarder, hexadecane 16% and 32% and dimethicon 25 cSt 32% were compared to MCT oil. The three additives’ viscosities were lower than pure MCT oil (Table 4). The thermodynamic activity of hexadecane 16% and 32% is around 0.7 and 0.6, assuming that the activity can be described using Raoult’s law, while both final conversions are around 0.83. Dimethicon 25 cSt 32% has a slightly higher conversion, about 0.92 (Table 2). However, the conversion speed (t_50%_) is within the same range as hexadecane 16% and 32% and dimethicon 25 cSt 32%. Figure 2 shows similar conversion curves between hexadecane 16% and 32%. Both curves have a slight decrease in the final conversion level, while little difference can be seen compared to the pure MCT oil before 50% conversion. However, when the relative conversion reaches 50%, half of the MCT oil has been hydrolyzed, but hexadecane maintains the same mass. Thus, the activity is expected to be even lower during the degradation process. Consequently, the observation is that the thermodynamic activity of the lipid does not seem to control the degradation rate.

### 3.3. Hypothesis 3: The Composition of the Interface Influences the Lipolysis

Four interfacial retarders (poloxamer 407, cetearyl glucoside, polysorbate 80, and monooleate) were evaluated to investigate if the composition of the oil–water interface during digestion could affect the lipolysis. The concentrations counted on the oil phase were lower (3%, 6%) than bulk retarders (16%, 32%). Concentrations counted on the water phase, ratios to bile salts, and interfacial tension of these interfacial retarders were measured and calculated to identify the key parameter influencing the interfacial composition. In vitro analysis and interfacial tension measurements were conducted to determine the correlation between the interfacial tension and retarding effect.

The thermodynamic activities of different formulations are similar because of the low concentration (Table 2). The interfacial tensions of all the measurable formulations, as well as pure bile extract (7 mN·m^−1^), pancreatin (10 mN·m^−1^), and the mixture of bile and pancreatin (4 mN·m^−1^) are all below 10 mN·m^−1^, which is relatively low.

**Figure 3:**
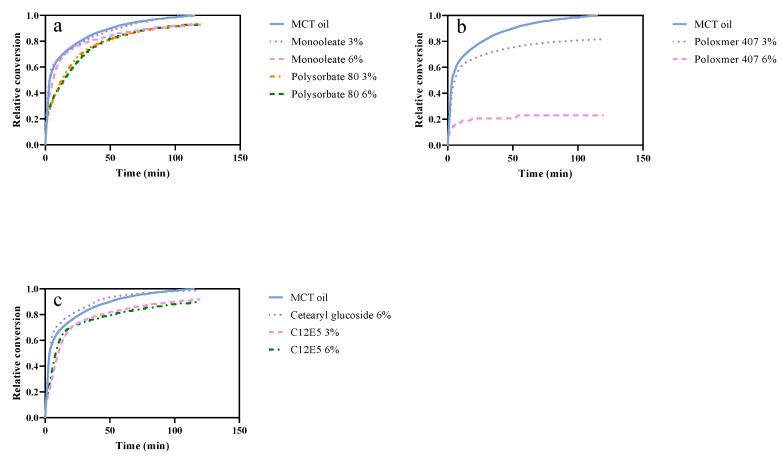
(**a**) Conversion of digestible interfacial retarders: monooleate 3%, and polysorbate, 3% and 6%, respectively. (**b**) Conversion of undigestible interfacial retarders: poloxamer 407 3% and 6%. (**c**) Conversion of undigestible interfacial retarders: cetearylglucoside 6%, C_12_E_5_ 3% and 6%.

From Figure 3, little difference in final conversion is observed among formulations having monooleate 3% and 6%, polysorbate 80 3% and 6%, or cetearylglucoside 6%. A slight decrease in conversion until 3000 s was seen when polysorbate 80 at concentrations of 3% and 6% were added into the formulation. However, the retardation is clearer when monitoring t_50%_, in Table 5, where polysorbate 80 and C_12_E_5_ (3% and 6%) need much more time to reach 50% conversion. Although the concentration of polysorbate 80 doubled from 3% to 6%, both conversion curves were quite similar and ended up with conversion of around 93% (Table 5). A significant retarding effect was observed when adding 6% poloxamer 407 into the formulation. In this case, less than 30% of the MCT oil was digested by pancreatic lipase. Thus, it is observed that interfacial additives may influence the degradation.

## 4. Discussion

Lipolysis is a heterogeneous reaction that occurs at the oil–water interface; therefore, the rate is influenced by adsorption and desorption of enzymatic reaction components, including substrates (oil molecules), products (FFAs), pancreatic lipase and colipase, bile salts, and retarders. Under in vivo conditions, there is a large surplus of enzymes and bile. Thus, in this work, we focused on the possibility of modifying the lipolysis rate by evaluating retarders inside the oil or localized at the surface of the oil droplets. The accessibility of the substrate from the oil phase can be divided into four parts: the diffusion of the oil molecule towards the interface (in liquid status), the thermodynamic activity of the oil phase, the status of the oil phase (liquid/semi-solid), and the interfacial composition that may prevent the lipase from adsorbing. The mechanisms are discussed based on the experiments using bulk and interfacial retarders. Meanwhile, chemical structures of retarders, digestibility of the retarders by pancreatic lipase, and possible retarding mechanisms are discussed.

### 4.1. Hypothesis 1: Change in the Consistency of the Oil Phase Influences the Lipolyze

It is expected that increasing the viscosity of the oil phase could decrease the flux of the oil molecules to the interface. Formulations containing PGPR 16% and 32% have viscosities around 2 and 5 times higher than pure MCT oil, respectively. However, both formulations have a similar conversion rate to pure MCT oil. PGPR is a substrate of pancreatic lipase, and it is gradually degraded during in vitro analysis. It seems that the viscosity of the oil phase has limited influence on lipolysis when using a digestible viscous retarder (PGPR). In the second experiment, an indigestible retarder, dimethicon, was used. Increased viscosity of the oil phase was obtained after adding a high-viscosity dimethicon (750 cSt) and by increasing the concentration of this retarder from 16% to 32%. The conversion after 2 h is slightly lowered to around 90% of the MCT oil, but the t_50%_ is increased to 2 min when increasing the viscosity 2–3-fold.

Ethylcellulose 300 cP 10%, on the other hand, has a strong retarding effect on lipolysis, as shown in Figure 1. The ethylcellulose forms a relatively firm and sticky gel, which traps the oil within the network. The diffusion of the oil molecules from inside the gel structure to the outside interface may be the key parameter controlling the lipolysis speed. Further investigations are needed, especially on the concentration and interaction of the polymer. However, the lower total interfacial area of ethylcellulose (total surface of side length of 2 mm cubes) compared to other retarders (total surface of diameter of 10 μm droplets) may contribute to the retardation.

To conclude, there is no linear correlation between the retarding effect and the viscosity. When the consistency of the oil phase is in the liquid state, an increase in the viscosity has a limited retarding effect. Meanwhile, the higher-concentration formulations (32%) do have a lower conversion speed which is indicated by more than doubled t_50%_. When the consistency of the oil phase is in semi solid-state, the retardation from the formed gel becomes significant.

### 4.2. Hypothesis 2: Thermodynamic Activity of Oil Phase Influences the Lipolysis

Thermodynamic activity is used to evaluate the speed of chemical reactions by recalculating the effective concentration of the mixture. In an enzymatic reaction such as lipolysis, the thermodynamic activity can also characterize the hydrolyze speed of the MCT oil. Because it is an intrinsic parameter that influences the conversion of MCT oil, thermodynamic activity of lipolysis can be applied to different retarders regardless of other retarding mechanisms.

The thermodynamic activity is estimated using Raoult’s law, approximating the solution of hexadecane in oil as being an ideal solution. Thus, the thermodynamic activity of the mixture is assumed to be proportional to the molar ratio:

(5) a≈X

By increasing the concentration of hexadecane from 16% to 32%, the approximated thermodynamic activity of MCT oil is assumed to decrease from about 0.7 to 0.6 compared to MCT oil alone. However, due to the differences in the nature of the molecules, the activity deviates from the ideal when the concentration of the additive increases. However, the similarities between conversion curves of pure MCT and hexadecane 16% and 32% at conversion levels less than 50% demonstrate that the thermodynamic activity is most likely not critical for the lipolysis rate. To further investigate the influence on lipolysis of thermodynamic activity, a formulation with dimethicon 25 cSt 32% was also compared. Like the formulations with hexadecane, the formulations with added dimethicon show a minor retarding effect during the in vitro analysis (longer t_50%_) until reaching the same endpoints of conversion (around 90%). This is an indication that thermodynamic activity changes the equilibrium level, while viscosity changes the kinetics of lipolysis.

### 4.3. Hypothesis 3: The Composition of the Interface Influences the Lipolysis

The “quality” of the interface has been long discussed in lipolysis and is believed to be the critical parameter that most influences lipolysis [51]. For example, it has been shown that the rate of lipolysis depends on the two-dimensional concentrations of enzymes and reactants at the interface rather than their concentrations in either of the bulk phases [52]. Bile salts at high concentrations reduce the biding capacity of lipase at the interface [52,53]. During lipolysis, pancreatic colipase binds to lipase in its active form and anchors it to the interface. In our system, the added surfactants might also influence the interface (including bile salts, lipase, colipase, and PC) during lipolysis.

Surfactants are known to outcompete proteins at the interface. For example, Wannerberg et al. [54] showed that lipase is competed out from a hydrophobic surface by C_12_E_5_ above the CMC. The experiments evaluating emulsifiers in our case included 3% and 6% monooleate, cetearylglucoside, polysorbate 80, poloxamer 407, and C_12_E_5_. These low concentrations resulted in similar viscosity and thermodynamic activity among all the formulations, exhibiting the precondition for discussing the correlation between retarding effects and the interface. Meanwhile, 3% and 6% are far above the CMC for the low molecular surfactant (CMC values shown in Table 2). Thus, the expectation is that the concentration of low-molecular-weight surfactants would have a lesser effect on the composition of the interface. This is also in agreement with results seen in Table 5.

The change in interfacial composition during lipolysis is a kinetic process. Therefore, the stability of the interface is a key parameter. As can be seen from the interfacial tension measurements, both the surfactants as such, the lipolysis system, and synergistically the surfactants together with the lipolysis have significantly interfacial tension lowering effects (Table 5). Thus, it is reasonable to assume that there could be a competitive strength of the retarders at the interface. Interfacial active retarders were added in considerable amounts above the CMC to ensure that the concentration is not limiting their ability to compete at the interface. The oil/water interfacial tension of the pure bile extract, pancreatin lipase, or the mixture of both is in the same range (less than 10 mN·m^−1^). The low interfacial tension of the mixture of bile and pancreatin (4 mN·m^−1^) also reveals the high affinity to the oil/water interface. Verger et al. [55] claimed that very low interfacial tension at the interface decreases the lipase activity because the lipase has less accessibility to the substrates. Gargouri et al. [56], on the other hand, concluded that no direct correlation between the decrease in lipase activity and the lowering of the interfacial tension could be observed. However, we observed (data not shown) that mixtures of surfactants and bile salt often decrease the surface tension even more than the two components individually, giving some indication that mixed-surface structures might occur.

Monooleate and polysorbate 80 are substances that can both be digested by lipase. Monooleate is a substrate of pancreatic lipase with an almost completed hydrolyzation. Thus, the conversion from monooleate is similar to MCT oil, both in t_50%_ and relative conversion. Polysorbate 80, on the other hand, can be hydrolyzed by lipase [57], but the digestion is expected be slower than for oil as well as the monoglyceride. In line with this, the t_50%_ for polysorbate 80 was longer than for oil and monoglyceride, but the final conversion level was more or less the same (Table 5).

To investigate if digestion is the only reason that low-molecular-weight surfactants had lesser effects on retardation, two non-digestible surfactants were investigated, C_12_E_5_ and cetearyl glucoside. Neither has a strong retarding effect. C_12_E_5_ has a weaker retarding effect than polysorbate 80, while cetearyl glucoside has no retarding effect at all. This indicates that for a retarder to have an efficient retarding effect, it being nondigestible by pancreatic lipase may have an influence, but it is not sufficient.

The lipolyze system investigated is a complex mixture of bile salt, retarding surfactants, and lipase. It is likely that the oil/waterinterface is also a complex mixture including bile salt, PC, and the surfactants added as retarders. Furthermore, the added surfactants can probably form mixed micelles with the bile, and we will have a kinetically controlled exchange of surfactants from the interface into the mixed bile salt–surfactant micelles in solution. The kinetics of this exchange could be one factor that affects the retarding capacity and especially the t_50%_ time. The structure of the interfacial layer could be another factor. It is interesting to note that it is the two PEG-based surfactants that have a reduction in the t_50%_ value. It is commonly accepted that PEG chains can present a steric hindrance, for example, for protein adsorption to interfaces. For example, Li and McClements [25] found that polysorbate 80 could decrease the release of the FFA from emulsions. The explanation proposed by Reis et al. [58] is that lower-molecular-weight surfactants (polysorbate 80) may expel proteins (pancreatic lipase) from the interface and thereby stop the lipolysis. This could explain why these surfactants initially have better retarding effects than the monoglyceride and the cetearyl glucoside.

Differing from the low-molecular-weight surfactants, the retarding effect of poloxamer 407 is largely related to its concentration in bulk (Figure 3b). It is strongly suspected that the composition of the interfacial layer changes when more poloxamer 407 is added which is also stated by others [59]. Porter et al. [60] and de Gennes [61] described the monolayer formation of PEO block as being “mushroom-type” when the concentration is low. When the concentration is comparatively high, PEO blocks will have stronger extensions into the water phase and form a “brush-type” conformation [48]. This conformational change reinforces the steric repulsion, preventing lipase from reaching the interface. There is also a hypothesis that “hemimicelle adsorption” may happen at the interface [62]. The same retarding effect of poloxamer 407 was obtained by other researchers both in vitro and in vivo [24,63,64]. Interestingly, the in vivo experiments show that P407 helps decrease the weight of mice by around 20% and that it reduces the plasma triglyceride concentration when the animals are exposed to a high-lipid diet [64].

## 5. Conclusions

In this work, we investigated different mechanisms for retardation of lipolysis. It was shown that increasing the viscosity or changing the thermodynamic activity of the system had only a minor retarding effect. However, using ethyl cellulose to form a stiff gel retarded the lipolysis considerably. Using interfacial retarders to hinder access of the lipase to the surface did only give a minor retardation when low molecular surfactants were used. However, a polymeric non-digestive surface-active retarder such as poloxamer 407, was able to form a steric layer that could significantly retard the lipolysis. Thus, both the change in the consistency of the oil phase and the exclusion of the lipase from the interface are possible mechanisms for retardation of lipolysis. However, the demands on the molecules used are extensive: in the first case the retardation is only seen when the molecules used can form an oleogel; in the latter case the surface-active component must have a strong irreversible adsorption to the interface.

## Figures and Tables

**Table 1:** The initial compositions of the simulated intestinal fluids.

Name	Reference Concentration *	Concentration after Modification
Bile extract	1.5–6.6 mmol/L	8 mmol/L
Phosphatidylcholine (soybean origin)	0	2 mmol/L
NaCl	150 mmol/L	150 mmol/L
Ca^2+^	4–12 mmol/L	15 mmol/L
Trizma-maleate	2 mmol/L	2 mmol/L
Pancreatin	270–1340 USP Unit	675 TBU
Total volume	300 mL	100 mL

* Data from [34].

**Table 3:** The viscosities of different formulations after pre-emulsification: dimethicon 25 cSt 16% and 32%, dimethicon 750 cSt 16% and 32%, PGPR 16% and 32%, ethylcellulose 300 cP, 10%.

Retarders	MCT Oil	Dimethicon 25 cSt, 16%	Dimethicon 25 cSt, 32%	Dimethicon 750 cSt, 16%	Dimethicon 750 cSt, 32%	PGPR, 16%	PGPR, 32%	Ethylcellulose 300 cP, 10%
Viscosity (mPa·s^−1^)	16	13	14	26	49–34	36	80	Too high to measure
Viscosity ratios to MCT oil	1	0.81	0.87	1.6	3.0–2.1 *	2.25	5	
Conversion (2 h) (φ) **	100%	98%	91%	92%	89%	98%	98%	38%
t_50%_ (min)	3.4	3.4	3.8	3.4	13.8	1.8	2.0	>120 min

* Retarders except for dimethicon 750 cSt show Newtonian character within the shear rate range of 10–100 s^−1^. Dimethicon 750 cSt is shear thinning. ** Standard error of mean of conversion data set (including bulk retarders and interfacial retarders) is 2%.

**Table 4:** Thermodynamic activity, final conversion (2 h), and the time point when reaching half final conversion (t_50%_) of hexadecane 16% and 32%, and dimethicon 25 cSt 32%.

Retarders	Hexadecane 16%	Hexadecane 32%	Dimethicon 25 cSt, 32%
Thermodynamic activity	0.73	0.58	N.A.
Viscosity ratio to MCT oil	0.69	0.48	0.87
Conversion (2 h) (φ)	83%	83%	92%
t_50%_ (min)	2.7	4.4	3.8

N.A. None applicable for dimethicon to usethe same thermodynamic activity estimation suggested for hexadecane. Standard error of mean of conversion data set (including bulk retarders and interfacial retarders) is 2%.

**Table 5:** Interfacial tension after the stability and conversion: monooleate, polysorbate 80, poloxamer 407, and cetearyl glucoside.

Retarders	Monooleate 3%	Monooleate 6%	Polysorbate 80, 3%	Polysorbate 80, 6%	Poloxamer 407, 3%	Poloxamer 407, 6%	C_12_E_5_ 3%	C_12_E_5_ 6%	Cetearyl Glucoside 6%
Interfacial tension after stability (mN·m^−1^)	N.A.	N.A.	4	4	6	6	<2 *	<2 *	N.A.
Conversion (2 h) (φ)	100%	92%	93%	93%	81%	23%	91%	90%	98%
t_50%_ (min)	3.1	6.0	12.6	14.5	5.4	>120 min	10	8.1	3.5

N.A. Nonapplicable. Monooleate and cetearylglucoside precipitated in the syringe. * The surface tension of C_12_E_5_ is lower than the detection limit; therefore, we assume it is less than 2 mN·m^−1^. Standard error of mean of conversion data set (including bulk retarders and interfacial retarders) is 2%.

## Supplementary Materials

The following supporting information can be downloaded at: https://www.mdpi.com/article/10.3390/pharmaceutics14050896/s1, Figure S1: Relative conversions of formulations with or without high-shear emulsification. (B). Relative conversions of formulations with a calcium concentration of 5 mM and 15 mM respectively; Figure S2: Size distribution (d(4,3)) after pre-emulsification of MCT oil, dimethicon 25 cSt 16%, dimethicon 25 cSt 32%, dimethicon 750 cSt 16%, dimethicon 750 cSt 32%, hexadecane 16%, hexadecane 32%, polysorbate 80 3%, polysorbate 80 6%, PGPR 16%, PGPR 32%, monooleate 3%, monooleate 6%, C_12_E_5_ 3%, C_12_E_5_ 6%.
