# Supplementary material for: Oil-Based Delivery Control Release System Targeted to the Later Part of the Gastrointestinal Tract—A Mechanistic Study
Source: Pharmaceutics. 2022 Apr 20;14(5):896. doi: 10.3390/pharmaceutics14050896 (PMC9144740; doi:10.3390/pharmaceutics14050896)
Supplement: Supplementary file 1 [file pharmaceutics-14-00896-s001.zip › pharmaceutics-1650730-supplementary.pdf]

Supply materials

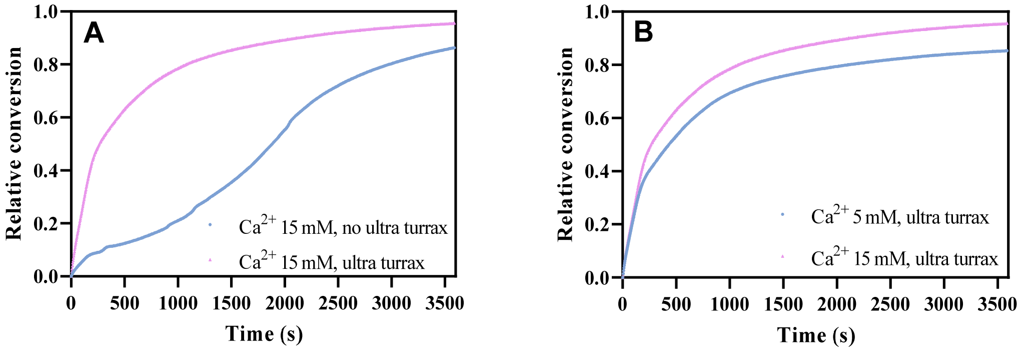

Figure S1. (A). Relative conversions of formulations with or without high-shear emulsification. **(B)**. Relative conversions of formulations with a calcium concentration of 5 mM and 15 mM respectively.

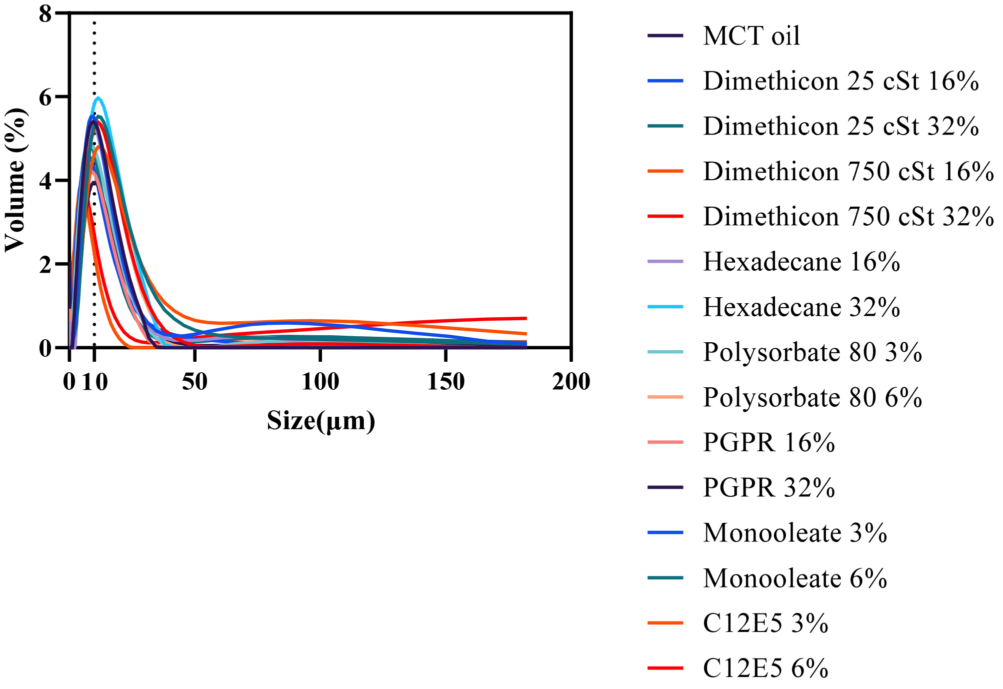

Figure S2. Size distribution (d(4,3)) after preeumilsification of MCT oil, Dimethicon 25 cSt 16%, Dimethicon 25 cSt 32%, Dimethicon 750 cSt 16%, Dimethicon 750 cSt 32%, Hexadecane 16%, Hexadecane 32%, Polysorbate 80 3%, Polysorbate 80 6%, PGPR 16%, PGPR 32%, Monooleate 3%, Monooleate 6%, C12E5 3%, C12E5 6%.
